# High-throughput format for the phenotyping of fungi on solid substrates

**DOI:** 10.1038/s41598-017-03598-9

**Published:** 2017-06-27

**Authors:** David Cánovas, Lena Studt, Ana T. Marcos, Joseph Strauss

**Affiliations:** 1 Division of Microbial Genetics and Pathogen Interaction, Department of Applied Genetics and Cell Biology, BOKU University of Natural Resources and Life Science, Campus Tulln, Tulln/Donau, Austria; 2 Departamento de Genética, Universidad de Sevilla, Seville, Spain; 3 Research Platform Bioactive Microbial Metabolites, BOKU University and University of Veterinary Medicine Vienna, Campus Tulln, Tulln, Austria; 4 Present Address: Instituto para el Estudio de la Reproducción Humana (Inebir). Avda de la Cruz Roja 1, 41009 Sevilla, Spain

**Keywords:** Reporter genes, High-throughput screening, Epigenetics, Fungal genetics

## Abstract

Filamentous fungi naturally grow on solid surfaces, yet most genetic and biochemical analyses are still performed in liquid cultures. Here, we report a multiplexing platform using high-throughput photometric continuous reading that allows parallel quantification of hyphal growth and reporter gene expression directly on solid medium, thereby mimicking natural environmental conditions. Using this system, we have quantified fungal growth and expression of secondary metabolite GFP-based reporter genes in saprophytic *Aspergillus* and phytopathogenic *Fusarium* species in response to different nutrients, stress conditions and epigenetic modifiers. With this method, we provide not only novel insights into the characteristic of fungal growth but also into the metabolic and time-dependent regulation of secondary metabolite gene expression.

## Introduction

The relevance of filamentous fungi for daily human life is immeasurable: fungi are drivers of biogeochemical nutrient cycles, producers of pharmaceuticals, cell factories for biotechnological applications and notorious pathogens of plants and animals. Although the majority of filamentous asco- and basidiomycetes is strictly aerobic and grows at the surface or inside solid substrates in their natural habitats, most of the experimental data have been obtained by growing these cells submerged in liquid media. The filamentous nature of most fungal species has hampered quantitative studies on solid substrates, and consequently the use of solid media usually is restricted to the isolation of single clones after mutagenesis or transformation, production of conidia as experimental starting material, or for phenotyping a small number of strains^1–5^. Yet, decades of research have made clear that the physiology and developmental biology of fungi substantially differs depending on whether they grow on a solid substrate or submerged in liquid media^1, 3, 6^. For example, some fungal isolates are good producers of enzymes or bioactive substances on solid but not in liquid media. A large number of plant pathogens infect their hosts through the surface of roots or shoots causing enormous losses in agriculture (e.g. *Fusarium*, *Magnaporthe*, *Ustilago*)^7–9^. Moreover, many filamentous fungi cannot even grow in submerged cultures^10^. The development of high-throughput technologies has allowed the swift analysis of a large number of samples in parallel, but yet high-throughput analysis of fungal cells is so far only performed for yeast but not filamentous fungi on solid substrates. Here, we present for the first time a scalable platform for multipurpose, 96-well plate format phenotyping of fungi on solid substrates in response to a diverse set of genetic or environmental cues.

## Results and Discussion

### Quantification of growth on solid media

**Figure 1:**
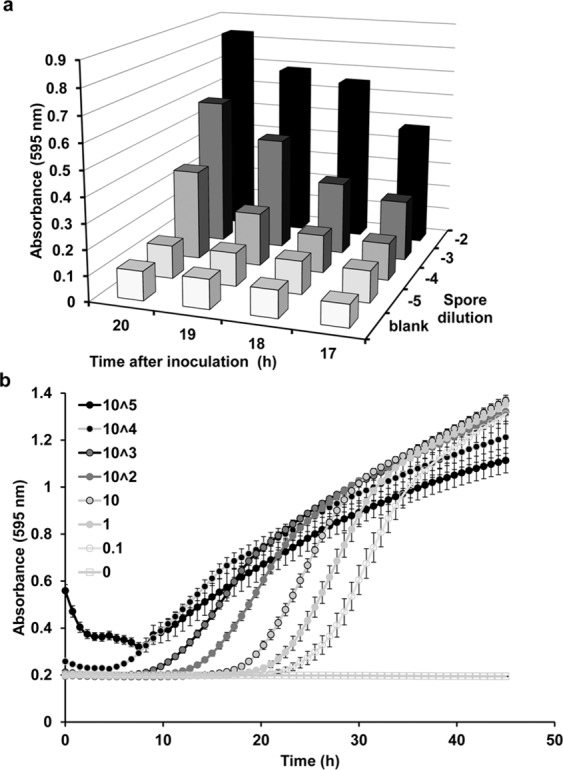
Quantification of fungal growth on solid media. (**a**) Initial test to determine if filamentous fungal growth on solid media can be monitored by measuring absorbance. Serial dilutions containing four different concentrations of spores of *A*. *nidulans* wild type were inoculated in the wells of a 96-well plate containing solid minimal media with ammonium as the sole nitrogen source. The plate was incubated at 37°C in an incubator. Absorbance was measured at 600 nm at 17 to 20 h post inoculation. We found that the absorbance of the culture was dependent on two parameters: time of growing incubation and the number of spores used for the inoculation. The blank controls (non-inoculated) showed a constant absorbance of 0.09–0.11, which did not change significantly along the experiment. Values shown are absolute values, and the blank controls were not subtracted from the readings. (**b**) Serial dilutions from 0.1 to 10^5^ spores/mm^2^ of the wild-type strain were inoculated in the wells containing solid minimal media, and growth was then monitored every 45 min at 37°C for 48 h. Three growth phases were clearly observed. The length of the lag phase was dependent on the initial inoculum (see Supplementary Fig. 2). Error bars represent the standard error of four independent experiments performed in quadruplicate.

Phenotyping individual fungal clones for growth characteristics such as biomass accumulation is routinely done by the tedious procedure of harvesting mycelium accumulated in submerged cultures and quantification of the dry weight, or ‘quick and dirty’ by measuring the radial extension of colonies on plates containing solid medium. Both methods do not provide an accurate reflection of the fungal growth as liquid media are not the natural substrates, and radial hyphal extension does not necessarily represent biomass accumulation (see below). Here, we report that the absorbance (optical density, OD) at 595 nm wavelength light can be used to monitor growth of *Aspergillus nidulans* and *Fusarium* species colonies growing on top of solid media, comparable to OD measurements of yeast and bacterial cells grown in liquid media. We tested the correlation of biomass accumulation with inoculation density and time of incubation for *A*. *nidulans* cells and found that both parameters correlated well with the number of spores inoculated per well and the time span of incubation (Fig. 1a). Distributional analysis of the OD readings performed over 25 squares per microtiter plate well revealed a Gaussian normal distribution of the signals over the colony with the highest OD at the colony center and lower ODs towards the edges of the colonies (Supplementary Fig. 1). It is therefore justified to suggest that OD_595_ measurements of colonies obtained with this system mirror their biomass formation. As biomass concentration changes over time represent growth, we have established here for the first time a platform that allows continuous automatic monitoring of filamentous fungal growth on solid medium, which can be easily scaled up to high-throughput. Three phases in the growth pattern became apparent (Fig. 1b). The length of the lag phase in which OD_595_ is at the level of background (non-inoculated control) inversely correlated with the inoculum size. We detected growth as early as nine hours after inoculation with 10^4^ spores/mm^2^ and as late as 27 hours after inoculation with 0.1 spores/mm^2^ (Supplementary Fig. 2). Spore concentrations in the range of 10^5^ spores/mm^2^ resulted in a strong background at the time of inoculation. During the following period the absorbance was increasing at maximum speed during aproximately 10 hours. At lower initial spore densities (0.1 to 100 spores/mm^2^) colonies showed the characteristics of an exponential growth phase, whereas higher spore densities above 10^3^ spores/mm^2^ led to a flattened growth curve indicating resource scantiness or mutual inhibition. The same mechanisms might account for the considerable attenuation of biomass accumulation at the end of the incubation period. Remarkably, the final biomass as measured by absorbance was similar for the different inoculation densities except for the highest 10^5^ spores/mm^2^ starting point, which resulted in the least biomass. Taken together, these characteristics indicate that colonies grow as long as resources are available but too high cell densities lead to growth inhibition, independently from resources.

### Influence of nutrient availability, auxotrophies and growth inhibitors

**Figure 2:**
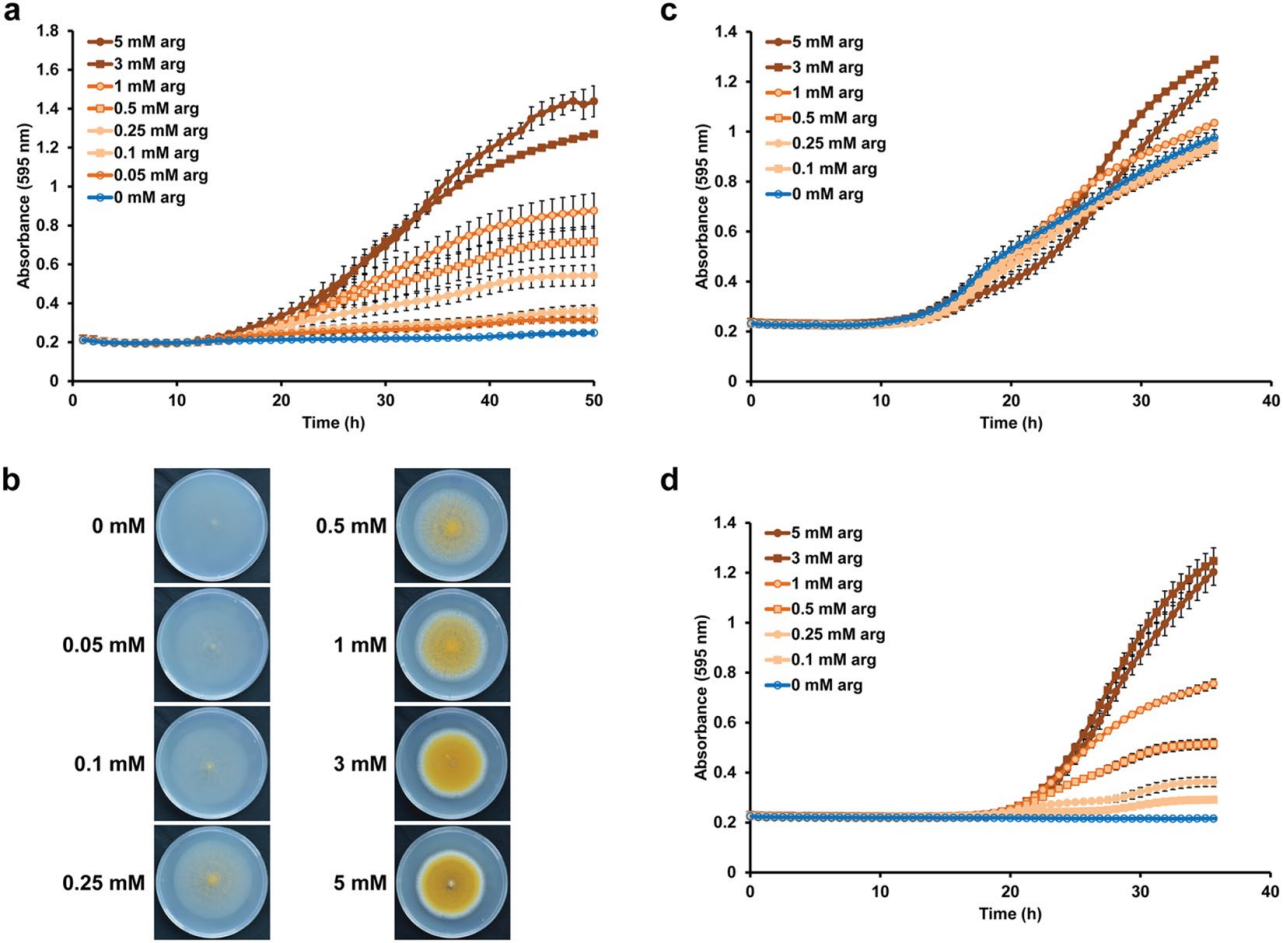
Effects of nutritional conditions or addition of antimicrobials. (**a**) 10^3^ spores/mm^2^ of the wild-type strain were inoculated in the wells containing solid minimal media with concentrations of arginine ranging from 0 to 5 mM. Arginine was employed as the sole nitrogen source in this experiment. (**b**) Conventional growth test of *A*. *nidulans* wild type on different concentrations of arginine as sole nitrogen source. Radial growth using solid media on petri dishes was the same regardless of the arginine concentration. However, it can be appreciated from the photographs that the density of the mycelial growth in the petri dishes was indeed dependent on the concentration of arginine. These differences in the mycelial density are hard to assess quantitatively using petri dishes. (**c**,**d**) The *A*. *nidulans* wild type (**c**) and the *argB2* mutant strain (**d**), which is auxotrophic for arginine, were inoculated on solid media containing ammonium as nitrogen source and different concentrations of arginine (0 to 5 mM) to supplement the auxotrophy. Error bars represent the standard error of two independent experiments performed in quadruplicate.

To test the influence of nutrients in more detail, one *A*. *nidulans* spore density (10^3^ spores/mm^2^) was employed to inoculate media with different concentrations of arginine, a well-suited nitrogen source. Both the speed of growth during the exponential phase and the maximum biomass the colony could reach were directly correlated with the nutrient concentration (Fig. 2a). Start of growth (lag phase) was not affected. Strikingly, the maximal radial extension of the colony was not dependent on the nutrient content and a test on conventional petri dishes clearly showed this effect as well (Fig. 2b). This demonstrates that biomass accumulation and radial colony diameters do not necessarily correlate, and direct biomass measurements (such as done here by OD_595_ absorbance) should be carried out. When arginine was employed as an auxotrophic supplement for the *argB2* mutant as well as the wild-type strain, only the growth of the *argB2* strain was dependent on the concentration of arginine (Fig. 2c,d). Notably, our findings match well with the concentration that is usually used to supplement the growth media of *Aspergillus* arginine-deficient mutants^11^.

**Figure 3:**
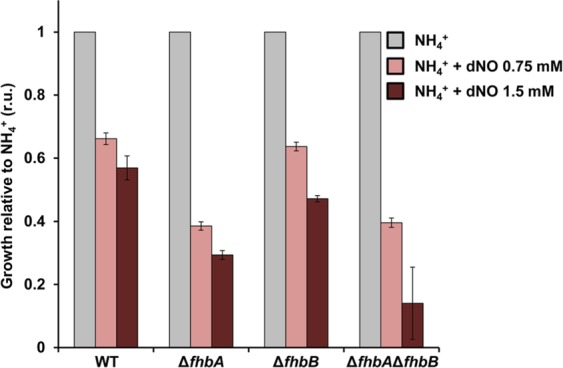
Quantitative assessment of the deleterious effects of nitrosative stress on the growth of *A*. *nidulans*. We employed the lag phase as an indicator of the growth of the culture and it was inversely normalised to the lag phase found in the wild type grown under control conditions (ammonium media in the absence of dNO). For an example of the complete growth curves, see Supplementary Fig. 3. Error bars represent the standard error of three independent experiments.

We tested this platform also for its suitability to monitor inhibition effects, e.g. by fungicides. This is a topic of major interest in medical mycology and agrobiotechnology. We have selected nitrosative stress, as a burst of nitric oxide (NO) has been shown to be involved in host defense mechanisms against some fungal pathogens^12^. Previously, we have tested the effects of commercially available compounds releasing NO on the wild type and a double mutant in the flavohemoglobin genes *fhbA* and *fhbB*, which are involved in the metabolisation of NO. However, commercially available drugs releasing NO, such as detaNONOate (dNO), are usually very expensive and only the double mutant in the flavohemoglobin genes was compared^13, 14^. Using our platform, here we have monitored growth of the wild type, of both the *fhbA* and the *fhbB* single mutants and of the double mutant (see an example of the growth curves of the wild type and double mutant in Supplementary Fig. 3). We employed the lag phase as an indicator of the growth of the culture and normalised it inversely to the lag phase found in the wild type grown under control conditions (ammonium media in the absence of dNO). Growth was reduced around 40% by addition of 1.5 mM dNO in the wild type that is able to detoxify NO through flavohemoglobins A and B, while the toxic effect at the same concentration is much more severe in a strain lacking both of the *fhbA fhbB* detoxification genes (Fig. 3). The Δ*fhbA* strain showed increased sensitivity to NO compared to the Δ*fhbB* mutant, which further confirms a main biological role for FhbA in nitrogen regulation of the cell, but not for FhbB. Thus, the platform applied here is able to accurately quantify the effect on growth. This may be important for future studies trying to quantify resistances or effects of fungicide combinations at nearly natural growth conditions.

### Multiplexing the platform to acquire fluorescent data during all growth stages

**Figure 4:**
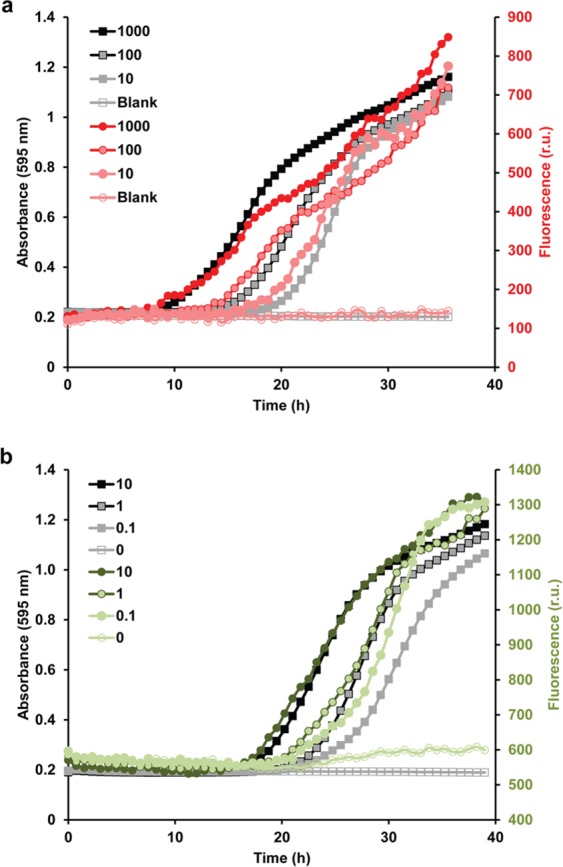
Multiplexing the platform to acquire quantitative growth and fluorescent data. Different conidial concentrations of the H1-RFP (**a**) and the gamma-actin-GFP (**b**) strains were inoculated on solid media containing ammonium as the sole nitrogen source. Growth was monitored by measuring absorbance at 595 nm (grey and black symbols). In parallel, fluorescence was detected as described in the Methods section (red symbols: fluorescence in the red channel; green symbols: fluorescence in the green channel).

In order to study whether this platform can be multiplexed for determining growth by OD_595_ and parallel fluorescent-based continuous reporter gene reading, we first tested two strains in which either histone H1 or gamma-actin were tagged with fluorescent proteins (H1-GFP and gamma-actin-RFP). In both cases, there was a good correlation between the increment in OD_595_ absorbance and fluorescence readings (Fig. 4a,b).

**Figure 5:**
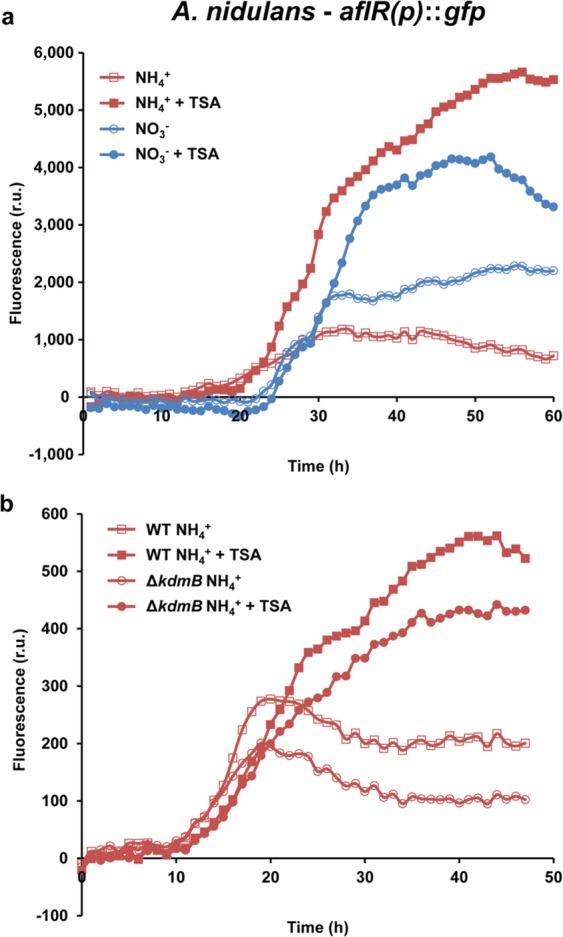
Hourly monitoring of the expression of the secondary metabolite genes during the entire fungal life cycle. Monitoring expression of *aflR*. (**a**) *A*. *nidulans aflR(p)::gfp* was grown on solid minimal media with ammonium (repressing) or nitrate (inducing) as sole nitrogen source. Media was supplemented with 0 µM or 1 µM trichostatin A (TSA), a histone deacetylase inhibitor known to increase SM gene expression of some SM gene clusters. (**b**) *A*. *nidulans aflR(p)::gfp* in a wild type (WT) or Δ*kdmB* mutant background were grown on solid minimal media with ammonium as sole nitrogen source at 37°C. Fluorescence and hence *aflR* expression is decreased upon deletion of *kdmB*. Addition of TSA increased the expression of *aflR* in both the wild type and the Δ*kdmB* mutant, as shown by an increase in the fluorescence readings. Growth and GFP fluorescence were monitored in parallel. Hyphal growth was only slightly affected upon deletion of *kdmB* as well as TSA treatment, as shown in the corresponding growth curves in Supplementary Fig. 4. The diagrams depict representative examples of the fluorescence patterns as the average from 3–4 samples.

Multiplexing would also allow to monitor gene regulation with reporter constructs in a time-dependent manner. We constructed reporter strains in which promoters of prominent secondary metabolite (SM)-encoding genes are fused to *gfp*, thereby allowing to monitor the expression of the respective gene as well as adjacent coregulated cluster genes. We chose to study the expression of biological relevant compounds, such as mycotoxins or virulence factors^15^. Each SM is transcribed only under specific environmental conditions. We thus wanted to perform a robust test of our platform to see whether also under these special conditions gene expression monitoring is possible. Therefore, the promoter of *aflR*, the narrow-domain transcription factor in the sterigmatocystin biosynthesis gene cluster was fused to *gfp* and stably integrated into the *A*. *nidulans* genome. On solid medium expression of *aflR* started already 20 h post inoculation (Fig. 5a), which is in agreement with known regulatory signals of development, but earlier than when the fungus grows in submerged cultures^16^. Again different to liquid media^16^, *aflR* expression was not completely repressed by ammonium. These differences show that gene expression analysis should also be performed under quasi-natural conditions when studying fungal physiology.

### Utilisation of the platform to monitor growth and gene expression in the plant-pathogenic fungi *F*. *graminearum* and *F*. *fujikuroi*

**Figure 6:**
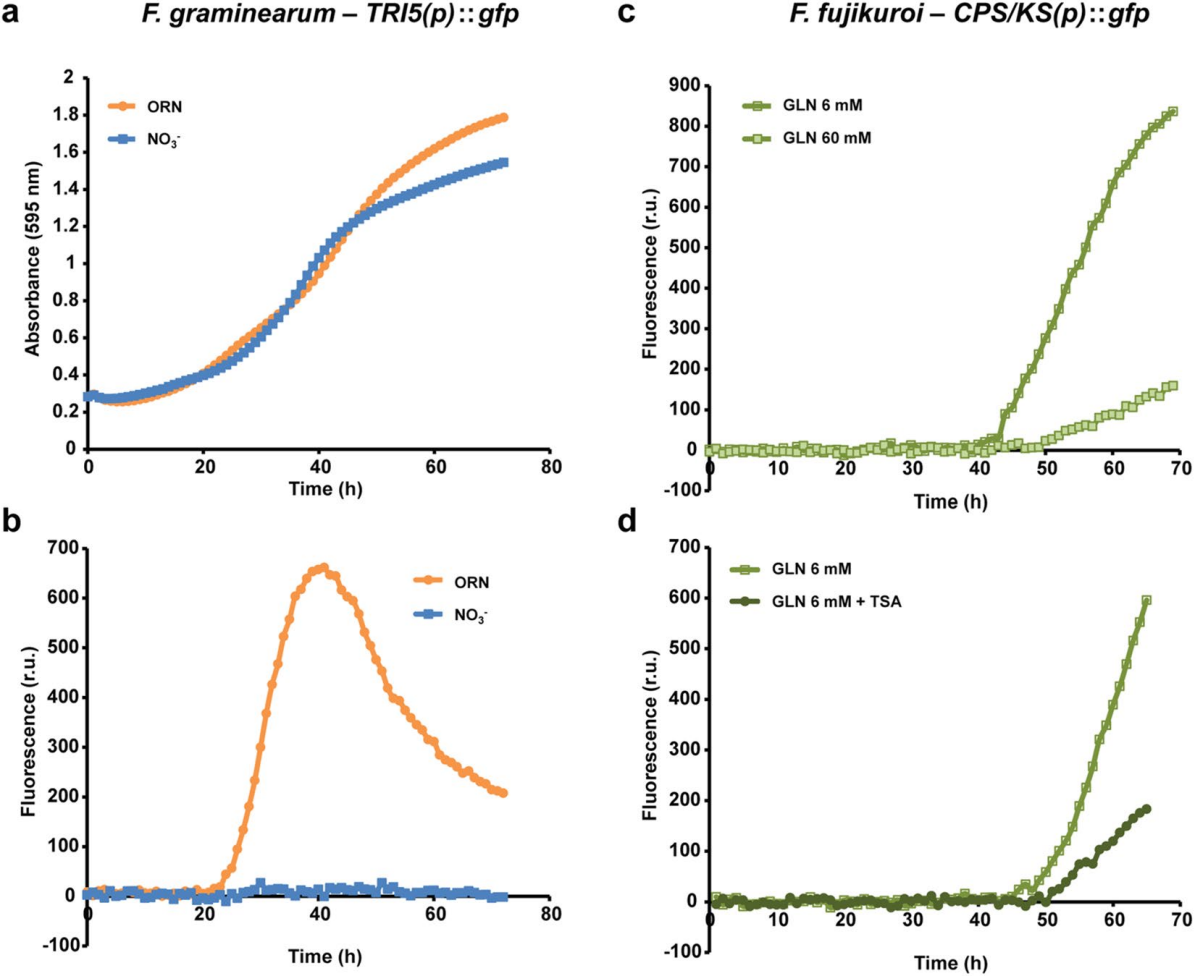
Monitoring expression of *TRI5*(*p*)*::gfp* and *CPS/KS*(*p*)*::gfp* in *F. graminearum* and *F. fujikuroi*. (**a**) The *F*. *graminearum* wild-type strain was grown on solid ﻿*Fusarium*﻿ minimal medium (FMM) with either 5 mM L-ornithine (ORN, inducing conditions) or 50 mM nitrate (NO_3_^−^, repressing conditions) at 24°C up to 72 h. Absorbance at 595 nm was monitored every hour. (**b**) In parallel, *TRI5* expression was quantified with the levels of GFP fluorescence. It was clearly detectable under inducing conditions, while fluorescence was not significantly detectable under repressing conditions. (**c**) The *F*. *fujikuroi* wild-type strain was grown on solid ICI medium with either 6 mM glutamine (GLN, inducing) or 60 mM glutamine (repressing) at 28°C up to 66 h. Fluorescence and hence *CPS/KS* expression is increased under inducing conditions compared to repressing conditions. (**d**) Addition of TSA to the culture media reduced the expression of *CPS/KS* grown under inducing conditions. Hyphal growth did not differ markedly between the different conditions (Supplementary Fig. 5). In all cases, data were acquired every one hour over the complete time course. The diagrams depict representative examples of the fluorescence patterns as the average from 3–4 samples.

Next, we wanted to extend our analysis to other filamentous fungi and thus tested the notorious plant pathogens *F*. *graminearum* and *F*. *fujikuroi*, a wheat and rice pathogen, respectively. In the case of these pathogens virulence is associated with the biosynthesis of specific SMs. For this, we fused *gfp* with promoters of signature genes involved in the biosynthesis of the two virulence factors deoxynivalenol (DON) and gibberellic acid (GA) produced by *F*. *graminearum* and *F*. *fujikuroi*, respectively^17, 18^. Similar to what we observed in *Aspergillus*, hyphal growth can be analysed reproducibly in both *Fusarium* species by OD_595_ measurements (Fig. 6a and Supplementary Fig. 5), demonstrating that this method is useful also for a variety of filamentous fungi. Reporter gene expression analysis confirmed earlier studies as biosynthesis of DON (*TRI5*(*p*)*::gfp*) and GA (*CPS*/*KS*(*p*)*::gfp*) was repressed by an alkaline pH and high nitrogen, respectively^19, 20^. In *F*. *graminearum*, 50 mM nitrate (high nitrogen and alkaline pH) repressed *TRI5* expression, and fluorescence was almost non-detectable, while it was induced by 5 mM ornithine up to 40 h post inoculation (Fig. 6b). Similarly, the *CKS/KS* expression was significantly higher when fluorescent-labelled strains were grown under nitrogen starvation (6 mM glutamine) compared to nitrogen excessive (60 mM glutamine) conditions (Fig. 6c). Hyphal growth was similar under both conditions for both fusaria (Fig. 6a and Supplementary Fig. 5).

### Monitoring of pharmacological and (epi)genetic alterations of gene expression

To test if physiological modulation by selected compounds is also possible in this system, we employed the histone deacetylase inhibitor trichostatin A (TSA), which is known to increase SM gene expression of some SM gene clusters^3, 21^. In agreement with previous data, treatment of the *aflR*(*p*)*::gfp* strain with TSA resulted in higher fluorescence both under inducing and non-inducing conditions (Fig. 5a) without affecting growth. Contrary to this, but in agreement with our previous results^20^, TSA treatment resulted in decreased *CPS/KS* gene expression in *F*. *fujikuroi* (Fig. 6d). Expression of *aflR* also depends on the H3K4 demethylase KdmB^16^. Indeed, an *aflR*(*p*)*::gfp* Δ*kdmB* strain showed lower expression levels of *aflR* than the wild type after 17–18 h of growth. However, only by continuous reading, we could now show for the first time that KdmB is involved in the maintenance of full expression, but not in triggering the signal because *aflR* expression started in both strains at the same time (Fig. 5b). In agreement with this hypothesis, addition of TSA boosted *aflR* expression in the Δ*kdmB* strain, but it did not achieve wild-type levels. Therefore, all our constructs perfectly mirror gene expression as it has been described earlier, thereby delivering a valuable tool to study effects on mycotoxin- or virulence factor-gene expression upon treatment with different supplements. Overall, the availability of this monitoring platform for virulence factor/SM gene expression upon treatment with e.g. different supplements or epigenetic modifiers provides new insights into the (epigenetic) regulation of secondary metabolite gene clusters.

## Conclusion

In conclusion, our platform enables for the first time the parallel quantitative analysis of many strains for growth on solid medium at very small time intervals in a potentially high-throughput format. This platform also allows for multiplexed measurements of growth and fluorescence-based reporter gene expression, which makes it very attractive for studying a range of other applications, such as the response to chemicals or environmental conditions. We can also envision further applications of this platform for high-throughput quantitative chemical genetics like screening individual clones of mutant libraries or compound libraries for effect on target gene expression.

## Methods

### Strains, media and culture conditions

*Aspergillus* and *Fusarium* strains used in this study are listed in Table S1. *Aspergillus* strains were grown at 37°C in minimal media containing the appropriate supplements^22^. 1% glucose was used as carbon source. Ammonium, nitrate and/or arginine were used as nitrogen sources as indicated. For protoplasting of *F*. *fujikuroi*, fungal strains were grown for 3 days in liquid Darken medium^23^ and 500 µL-aliquot of this pre-culture was taken and transferred into 100 mL synthetic ICI medium^24^, that contained 10 g/L fructose instead of glucose and 1 g/L (NH_4_)_2_SO_4_ as nitrogen source. Strains were incubated on a rotary shaker at 28°C and 180 rpm no longer than 16 h. For protoplasting of *F*. *graminearum*, YPG media was inoculated with approximately 10^6^ conidia and incubated overnight on a rotary shaker at 30°C and 180 rpm. For conidiation, *F*. *graminearum* strains were grown for seven days in liquid carboxymethylcellulose media (CMC) on a rotary shaker at 24°C and 180 rpm^25^. In case of *F*. *fujikuroi*, solid vegetable juice (V8) medium was inoculated with 5 mm agar plaques and incubated for seven days at 20°C and 12 h/12 h light-dark cycle. DNA isolation was performed on fungal strains grown on solid complete medium (CM)^11^ covered with cellophane sheets for three days in constant darkness at 30°C in case of *F*. *fujikuroi* or 20°C in case of *F*. *graminearum*. For expression analyses, conidia were inoculated in solid ICI medium with either 6 mM or 60 mM glutamine as sole nitrogen source in case of *F*. *fujikuroi*, and in solid *Fusarium* minimal medium^26^, with either 5 mM sodium nitrate or 50 mM L-ornithine as sole nitrogen source in case of *F*. *graminearum*.

### Chemicals

Trichostatin A and detaNONOate were obtained from Sigma and used at the indicated concentrations.

### Plasmid constructions

Plasmids for *A. nidulans*, *F. fujikuroi* and *F. graminearum* fluorescent strains were generated using yeast recombinational cloning^27^. All primers used for polymerase chain reaction (PCR) are listed in Table S2 and were obtained from Sigma-Aldrich. In case of *A*. *nidulans,* the upstream (5′) and downstream (3′) sequences of *aflR* was amplified from *A. nidulans* genomic DNA using the following primer pairs: YRC_*aflR*_5F and YRC_*aflR*-GFP_R for upstream and YRC_*aflR*_3F and YRC_alfR_3R for downstream regions. The reporter gene *gfp* and the auxotrophy marker *riboB* were amplified using the primer pairs GFP-F and Tgluc-RIBO_R and pRIBO-F and tRIBO-R, respectively. In case of *F*. *fujikuroi* and *F*. *graminearum*, the upstream (5′) and downstream (3′) sequences of *CPS/KS* and *TRI5* were amplified from *F. fujikuroi* IMI58289 and *F. graminearum* PH-1 genomic DNA, respectively, using the following primer pairs: YRC_CPS/KS_5F and YRC_CPS/KS-GFP_R for upstream and YRC_CPS/KS_3F and YRC_CPS/KS_3R for downstream regions in case of *F. fujikuroi* and YRC_TRI5_5F and YRC_TRI5-GFP_R for upstream and YRC_TRI5_3F and YRC_TRI5_3R for downstream regions in case of *F. graminearum*. The fragment containing the reporter gene *gfp* and an artificial terminator sequence, that is, *Tgluc* from *Botrytis cinerea*, and the resistance cassette hygromycin B, *hph*, driven by the *trpC* promoter was amplified from p*bcltf3-*GFP﻿^﻿﻿iL^ (J. Schumacher, unpublished)﻿﻿ using the primer pair gfp-F and hph-F.

The *S. cerevisiae* strain FY834 was co-transformed with the obtained fragments and the *Eco*RI/*Xho*I-restricted plasmid pRS426^27^. Correct assembly of the resulting plasmids was verified by sequencing.

### Construction of strains

Construction of *A*. *nidulans* strains was done following standard methods^28, 29^. In the constructs, the *gfp* gene replaces the ORF of *aflR* and its expression is under the control of the corresponding promoters, respectively. Consequently, the strains harboring the *gfp* fusions are *∆aflR*. Homologous integration of the *aflR*(*p*)::*gfp* construct was verified by diagnostic PCR using the primer pairs dPCR_IL_aflRup_5F and dPCR_IL_GFP-out-R, and dPCR_IL_RIBOout_R and dPCR_IL_aflRdown_3. The number of copies of the construct integrated in the genome was checked by qPCR, and only strains harboring one copy were used for further experiments.

For generation of *F. fujikuroi* and *F*. *graminearum* mutants, protoplasts were prepared from the wild-type strains IMI58289 and PH-1, respectively. Transformations were carried out as previously described^30^. In case of *F. graminearum*, we used the split-marker approach^31^. For this, the replacement fragment was amplified in two PCRs containing about two thirds of the resistance cassette, *hph*, each from p*TRI5(p)::gfp* using the primer pairs YRC_TRI5_5F and hyg_split-mark_F as well as hyg_split-mark_R and YRC_TRI5_3R. Transformation of *F. graminearum* was according to *F. fujikuroi* with minor adjustments. All transformed protoplasts were regenerated as described^32^. Single conidial cultures were established from either hygromycin B-resistant transformants and used for subsequent DNA isolation. Homologous integration of the GFP construct was verified using the primer pairs GGS2-D-DR2 and ogfp_seqR1.

### Plate reader methods

All experiments were performed in 96-wells plates containing 100 µL of solid media with the appropriate supplements and the indicated nitrogen source per well. TSA or detaNONOate was added to melted media just before being dispensed into the wells. Plates were inoculated with 30–40 µL of different concentrations of conidia as indicated and incubated at 37°C (*A*. *nidulans*), 28°C (*F*. *fujikuroi*) or 24°C (*F*. *graminearum*) in the plate reader. Absorbance and fluorescence was detected and quantified at the indicated time intervals (usually 45–60 minutes) in a Synergy HT or a Synergy Mx Multi-mode Microplate Readers (Biotek) equipped with GFP (Ex 485/Em 528) and RFP (Ex 580/Em 610) filter sets. Absorbance was quantified at 595 nm (Synergy HT) or 540 nm (Synergy Mx) depending on the filters available at the microplate reader employed. Blanks were regularly inoculated with water. In the experiments involving fluorescence, a non-fluorescent wild type strain was employed to subtract the background fluorescence from the sample data. Data were recorded and analysed with Gen5^TM^ Data Analysis Software v2.0 and exported to Microsoft Excel for further analysis and generation of the graphs. Lag times were calculated using Gen5^TM^ Data Analysis Software v2.0 as the time interval between the line of maximum slope of the propagation phase and the absorbance baseline at time zero. Experiments were repeated at least two times in duplicates to quadruplicates.

## Electronic supplementary material

Supplementary Information
