# Author Correction: High-throughput format for the phenotyping of fungi on solid substrates

**DOI:** 10.1038/s41598-020-61396-2

**Published:** 2020-03-06

**Authors:** David Cánovas, Lena Studt, Ana T. Marcos, Joseph Strauss

**Affiliations:** 1 Division of Microbial Genetics and Pathogen Interaction, Department of Applied Genetics and Cell Biology, BOKU University of Natural Resources and Life Science, Campus Tulln, Tulln/Donau, Austria; 2 Departamento de Genética, Universidad de Sevilla, Seville, Spain; 3 Research Platform Bioactive Microbial Metabolites, BOKU University and University of Veterinary Medicine Vienna, Campus Tulln, Tulln, Austria; 4 Present Address: Instituto para el Estudio de la Reproducción Humana (Inebir). Avda de la Cruz Roja 1, 41009 Sevilla, Spain

Correction to: *Scientific Reports* 10.1038/s41598-017-03598-9, published online 27 June 2017

In the Supplementary Information file originally published with this Article, additional datasets detailing the fungal strains and genetic primers used in this study were unintentionally omitted. These errors have been corrected in the Supplementary Information that now accompanies the Article.

