# Supplementary material for: High-throughput format for the phenotyping of fungi on solid substrates
Source: Sci Rep. 2017 Jun 27;7:4289. doi: 10.1038/s41598-017-03598-9 (PMC5487330; doi:10.1038/s41598-017-03598-9)
Supplement: Supplementary file 1 — Supplementary Information [file 41598_2017_3598_MOESM1_ESM.pdf]

SUPPLEMENTARY INFORMATION

High-throughput format for the phenotyping of fungi on solid substrates

David Cánovas, Lena Studt, Ana T. Marcos, and Joseph Strauss

Supplementary Figures Legends

Supplementary Fig. 1. In-well analysis of the distribution of the signal. A grid of 5x5 squares was designed and absorbance values were individually quantified at each of the 25 positions. The signal is uniformly distributed in the center of well and showing lower values at the border of the wells, which correspond with areas shared between the inside of the well and the walls. This experiment was performed in an EnSpire Multimode plate reader (Perkin Elmer).

Supplementary Fig. 2. Correlation between inoculum size and lag phase. Serial dilutions of spores were inoculated on minimal solid media containing ammonium as the sole nitrogen source and incubated at 37 °C in the plate reader. Absorbance was recorded for 48 h. The lag phase was plotted against the logarithmic concentration of spores in the inoculum, and the trend line was calculated. The data shows an inverse logarithmic correlation with a coefficient of determination (R^2) of 0.994. Data is the average of four independent experiments performed at least in duplicates. Error bars depict the standard error of the sample.

Supplementary Fig. 3. Quantitative assessment of the deleterious effects of nitrosative stress on the growth of *A. nidulans*. Using our platform here we have quantified growth of the wild type and the double mutant. The plot displays the growth curves of both strains in ammonium minimal media containing 0, 0.75 or 1.5 mM dNO. Both strains displayed the same growth pattern in the absence of dNO. Addition of dNO to the media increased the length of the lag phase, and this increase was more drastic in the case of the double mutant, *ΔfhbA ΔfhbB*, that hardly grew in the presence of 1.5 mM dNO. The experiment also included both single mutants (not depicted here for clarity). We employed the lag phase as an indicator of the growth of the culture and it was inversely normalized to the lag phase found in the wild type grown under control conditions (ammonium media in the absence of dNO). The experiment was repeated three times, although only one experiment is represented here as an example. Figure 3 contains the calculations of the three independent experiments and errors bars depicting the standard error of each sample.

Supplementary Fig. 4. Growth curves corresponding to the samples in Fig. 5b.

Supplementary Fig. 5. Growth curves corresponding to the samples in Fig. 6c-d.

Table S1

Strains used in this study

A. nidulans strains	Genotype[*]	Reference
Cib08	*biA1 yA2 veA1*	[14]
	argB2 biA1 veA1	FGSC[¶]
	pyroA4 veA1	FGSC
TNO2A21	*riboB2 pyroA4 nku::argB veA1*	[28]
CibA	*ΔfhbA biA1 yA2 veA1*	[14]
CibB	*ΔfhbB biA1 yA2 veA1*	[14]
CibAB	*ΔfhbA ΔfhbB biA1 yA2 veA1*	[14]
Ag07C1#7	*ΔkdmB::AfriboB ΔhepA::AfpyrG pabaB22 pantoB100 ΔniaD Δnku::argB(?)*	[16]
aflR(p)::egfp	*aflR(p)::egfp::afriboB::(ΔaflR) riboB2 pyroA4 Δnku::argB veA1*	This study
aflR(p)::egfp Δ*kdmB*	*aflR(p)::egfp::afriboB::(ΔaflR) kdmBΔ::AfriboB riboB2(?) ΔniaD Δnku::argB(?) veA1*	This study
	gamma-actin::gfp pyroA4 wA3	From Claudio Scazzocchio
	H1::rfp pabaA1 wA3	From Claudio Scazzocchio
F. fujikori strains		
TRI5(p)::egfp	*TRI5(p)::egfp, hph, ΔTRI5*	This study
CPS/KS(p)::egfp	*CPS/KS(p)::egfp, hph, ΔCPS/KS*	This study

[*] All strains are *veA1*

[¶] Fungal Genetics Stock Center (Department of Plant Pathology, 4024 Throckmorton, Plant Sciences Center, Kansas State University, Manhattan, KS 66506, USA)

Table S2

Primers used in this study.

Oligo	Sequence
YRC_CPS/KS_5F	TAACGCCAGGGTTTTCCCAGTCACGACGCTTCCACCTGTGATTGAGCTCC
YRC_CPS/KS-GFP_R	TACTTACCTCACCCTTGGAAACCATCTTGTGTAAGTATTCCAGATCCAC
YRC_CPS/KS_3F	TCCTTCAATATCATCTTCTGTCTCCGACAGGGAATACTATGGAGCTGG
YRC_CPS/KS_3R	GCGGATAACAATTTCACACAGGAAACAGCGAATCTCAGGTCTGATGGTGG
YRC_TRI5_5F	TAACGCCAGGGTTTTCCCAGTCACGACGGACGGCAGACAGCGGCTATACG
YRC_TRI5-GFP_R	TACTTACCTCACCCTTGGAAACCATGATGGCAAGGTTGTACTGG
YRC_TRI5_3F	TCCTTCAATATCATCTTCTGTCTCCGACGATACTCGTTTGGAGAATGGTGG
YRC_TRI5_3R	GCGGATAACAATTTCACACAGGAAACAGCTCGAGCATCGACATCGAGCATCG
gfp-F	ATGGTTTCCAAGGGTGAGG
hph-F	GTCGGAGACAGAAGATGATATTGAAGGAGC
YRC_*aflR*_5F	TAACGCCAGGGTTTTCCCAGTCACGACGAAGCGAAGCACCTCATCAACGG
YRC_*aflR*-GFP_R	TACTTACCTCACCCTTGGAAACCATGATATTTGCATATGATACAGG
YRC_*aflR*_3F	GGTGTATGCTCGTCACACTCATGACGAGTGTTGGGTGAATGG
YRC_alfR_3R	GGATAACAATTTCACACAGGAAACAGCTCTCCGCAATCGCACTTCGCAGG
Tgluc-RIBO_R	AGCCAGACTCCTGAACGGCCTCATCTTGTTGGGGGGAAGGGG
pRIBO-F	GAGGCCGTTCAGGAGTCTGGCTCG
tRIBO-R	CCATTCACCCAACACTCGTCATGAGTGTGACGAGCATACACC
hyg_split-mark_F	GGATGCCTCCGCTCGAAGTA
hyg_split-mark_R	CGTTGCAAGACCTGCCTGAA
GGS2-D-DR2	TGATCACCGAGGTCTCGTCTTCGG

ogfp_seqR1	CGTCTCCCTCACCCTCTCCG
pCSN44-trpC-P2	GTGATCCGCCTGGACGACTAAACC
Seq-P450-3R	AGCCTATGGAAGCTTGTAGC
pCSN44-trpC-P	CCTCCACTAGCTCCAGCCAAGCCC
TRI5-downstream_R	CTCCGACGTATTCGAAGC
dPCR_IL_*aflR*up_5F	GCTGACATCCACGATGGTGG
dPCR_IL_GFP-out-R	CGACCAAGATTGGAACGACGC
dPCR_IL_RIBOout_R	GACTTAGCCAAGTCGACACG
dPCR_IL_*aflR*down_3R	GACGGTCTACCTAGTGACCG

Supplementary Figure 1

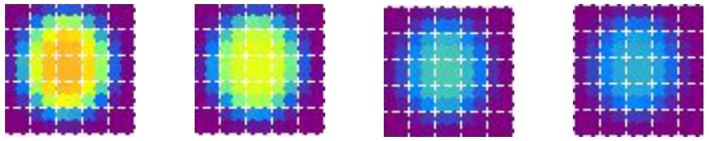

Supplementary Fig. 1. In-well analysis of the distribution of the signal. A grid of 5x5 squares was designed and absorbance values were individually quantified at each of the 25 positions. The signal is uniformly distributed in the center of well and showing lower values at the border of the wells, which correspond with areas shared between the inside of the well and the walls. This experiment was performed in an EnSpire Multimode plate reader (Perkin Elmer).

Supplementary Figure 2

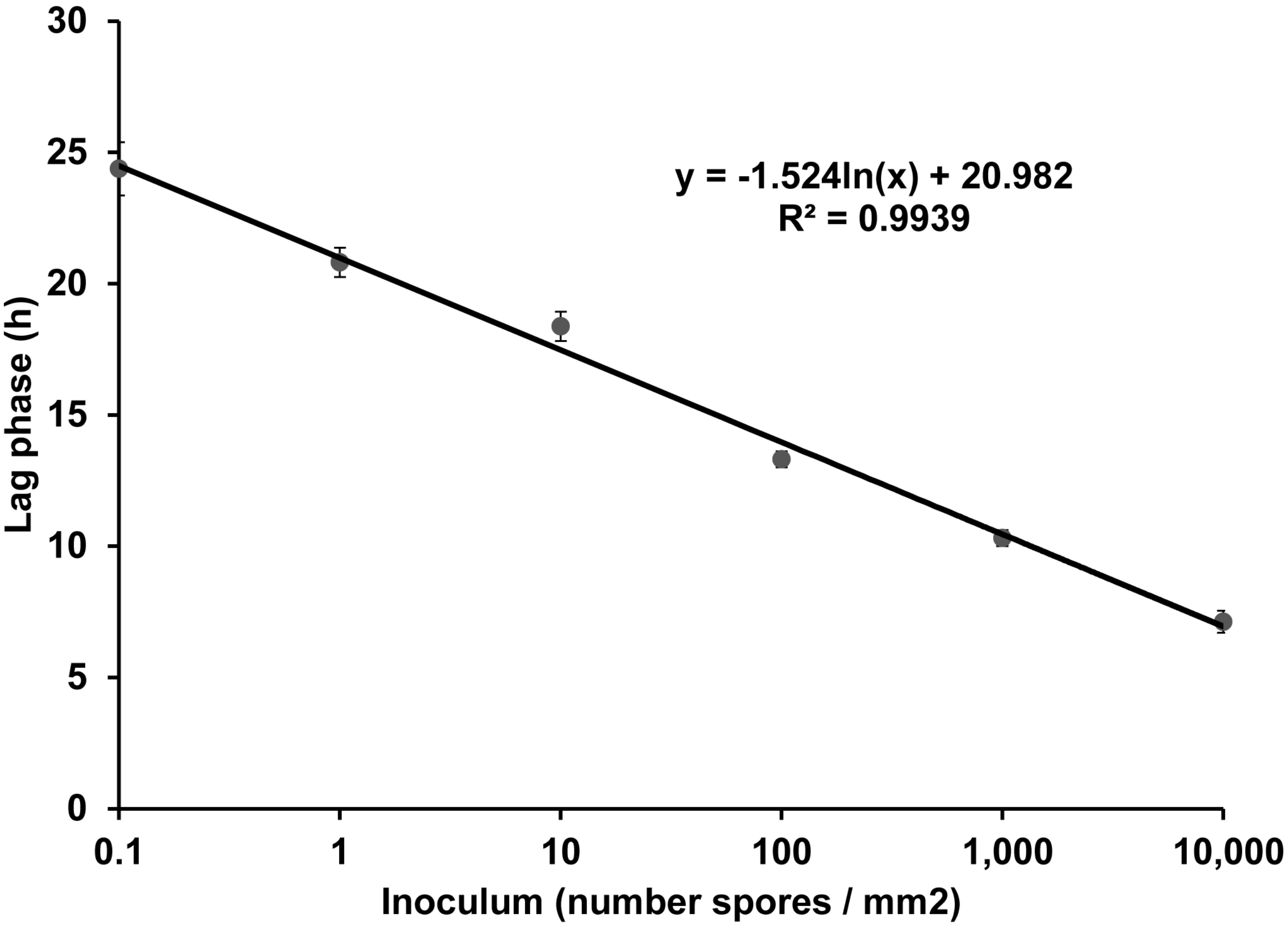

Supplementary Fig. 2. Correlation between inoculum size and lag phase. Serial dilutions of spores were inoculated on minimal solid media containing ammonium as the sole nitrogen source and incubated at 37 °C in the plate reader. Absorbance was recorded for 48 h. The lag phase was plotted against the logarithmic concentration of spores in the inoculum, and the trend line was calculated. The data shows an inverse logarithmic correlation with a coefficient of determination (R^2) of 0.994. Data is the average of four independent experiments performed at least in duplicates. Error bars depict the standard error of the sample.

Supplementary Figure 3

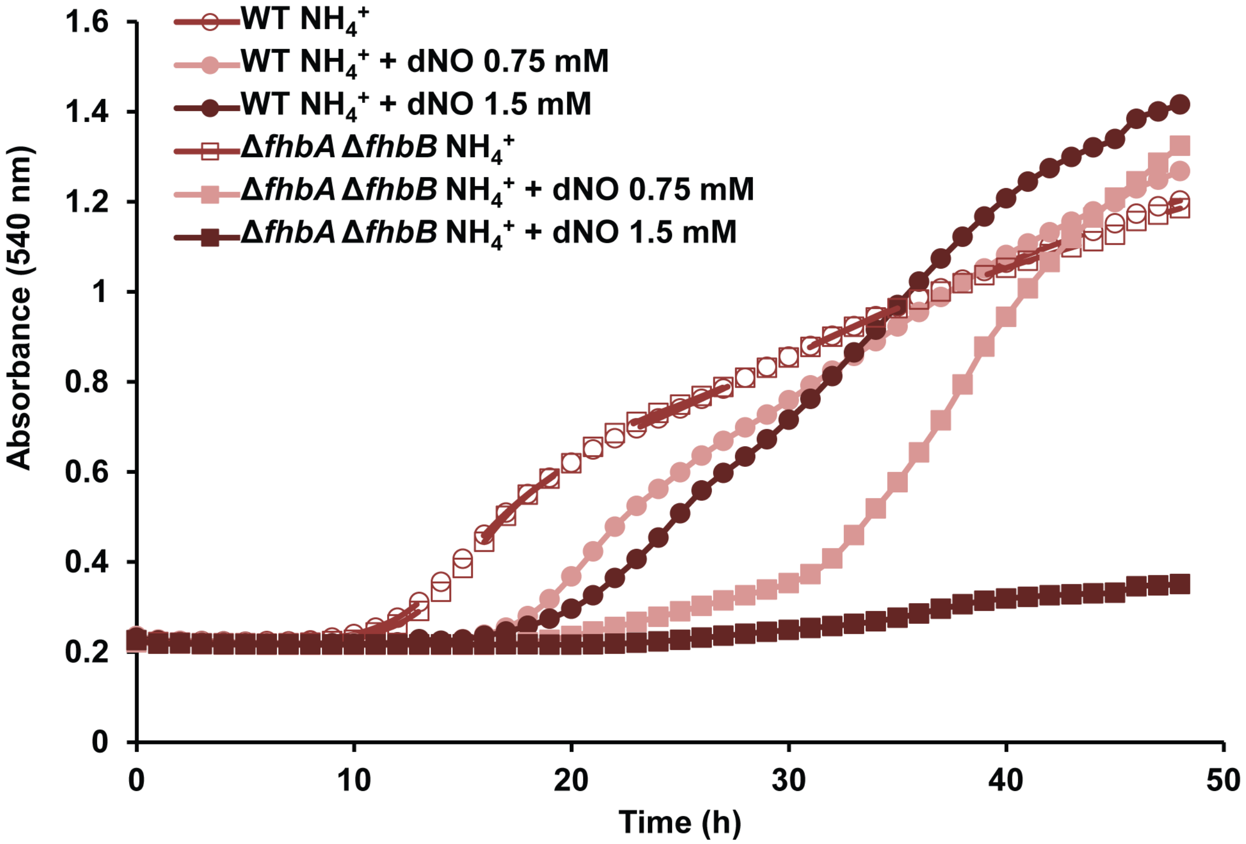

Supplementary Fig. 3. Quantitative assessment of the deleterious effects of nitrosative stress on the growth of *A. nidulans*. Using our platform here we have quantified growth of the wild type and the double mutant. The plot displays the growth curves of both strains in ammonium minimal media containing 0, 0.75 or 1.5 mM dNO. Both strains displayed the same growth pattern in the absence of dNO. Addition of dNO to the media increased the length of the lag phase, and this increase was more drastic in the case of the double mutant, *ΔfhbA ΔfhbB*, that hardly grew in the presence of 1.5 mM dNO. The experiment also included both single mutants (not depicted here for clarity). We employed the lag phase as an indicator of the growth of the culture and it was inversely normalized to the lag phase found in the wild type grown under control conditions (ammonium media in the absence of dNO). The experiment was repeated three times, although only one experiment is represented here as an example. Figure 3 contains the calculations of the three independent experiments and errors bars depicting the standard error of each sample.

Supplementary Figure 4

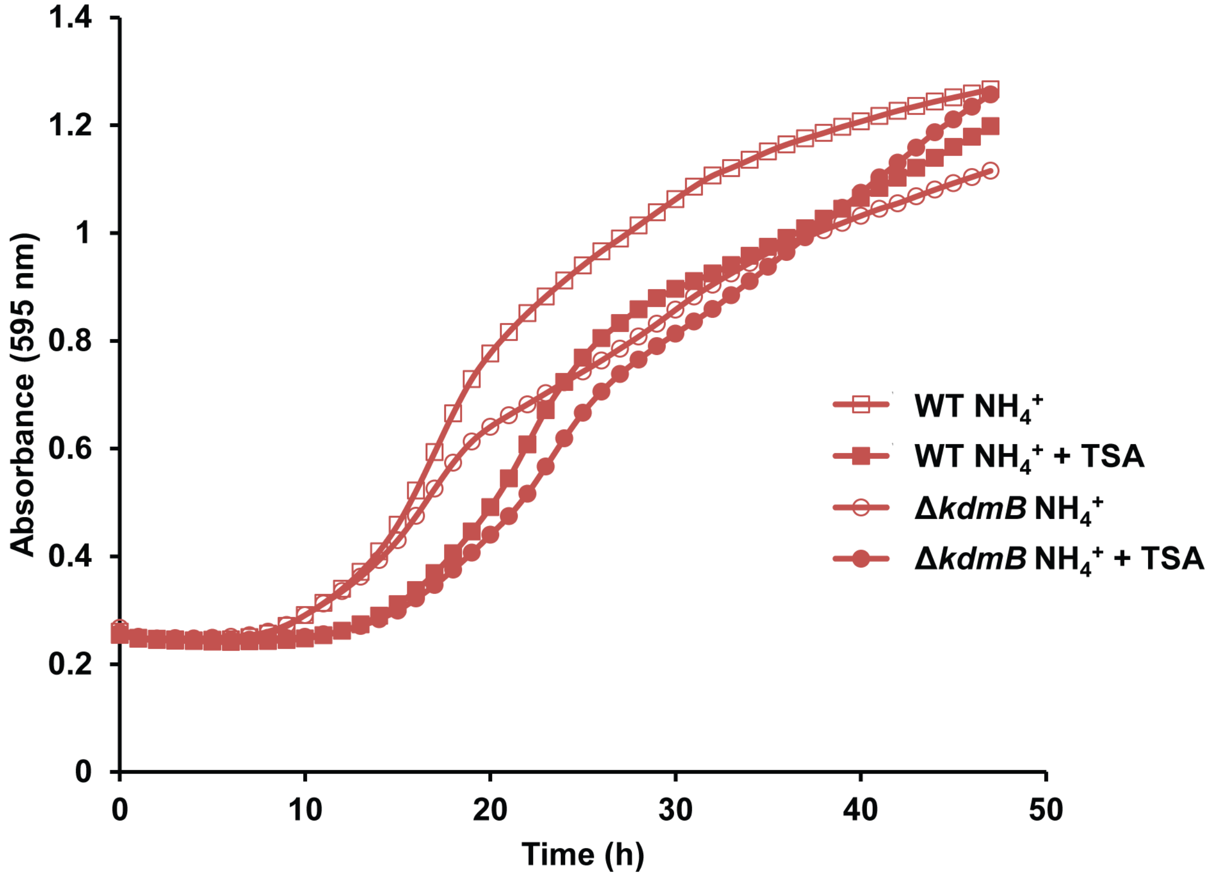

Supplementary Fig. 4. Growth curves corresponding to the samples in Fig. 5b.

Supplementary Figure 5

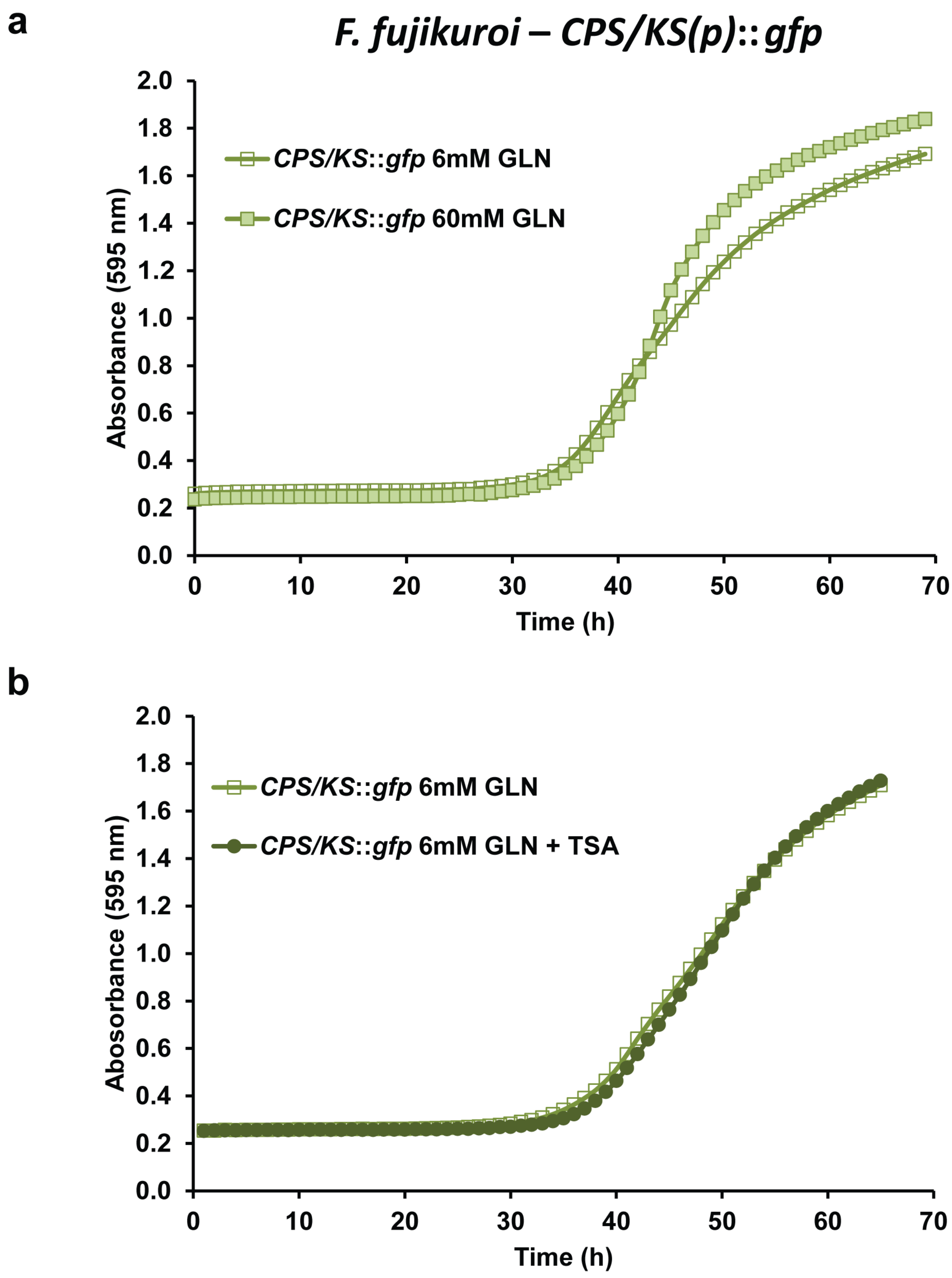

Supplementary Fig. 5. Growth curves corresponding to the samples in Fig. 6c-d.
